# Enantioseparation of Citalopram by RP-HPLC, Using Sulfobutyl Ether-*β*-Cyclodextrin as a Chiral Mobile Phase Additive

**DOI:** 10.1155/2016/1231386

**Published:** 2016-01-05

**Authors:** Yangfeng Peng, Quan Sophia He, Jiang Cai

**Affiliations:** ^1^School of Chemical Engineering, East China University of Science and Technology, Shanghai 200237, China; ^2^Department of Engineering, Faculty of Agriculture, Dalhousie University, Truro, NS, Canada B2N 5E3

## Abstract

Enantiomeric separation of citalopram (CIT) was developed using a reversed phase HPLC (RP-HPLC) with sulfobutylether-*β*-cyclodextrin (SBE-*β*-CD) as a chiral mobile phase additive. The effects of the pH value of aqueous buffer, concentration of chiral additive, composition of mobile phase, and column temperature on the enantioseparation of CIT were investigated on the Hedera ODS-2 C_18_ column (250 mm × 4.6 mm × 5.0 um). A satisfactory resolution was achieved at 25°C using a mobile phase consisting of a mixture of aqueous buffer (pH of 2.5, 5 mM sodium dihydrogen phosphate, and 12 mM SBE-*β*-CD), methanol, and acetonitrile with a volumetric ratio of 21 : 3 : 1 and flow rate of 1.0 mL/min. This analytical method was evaluated by examining the precision (lower than 3.0%), linearity (regression coefficients close to 1), limit of detection (0.070 *µ*g/mL for (*R*)-CIT and 0.076 *µ*g/mL for (*S*)-CIT), and limit of quantitation (0.235 *µ*g/mL for (*R*)-CIT and 0.254 *µ*g/mL for (*S*)-CIT).

## 1. Introduction

**Figure 1:**
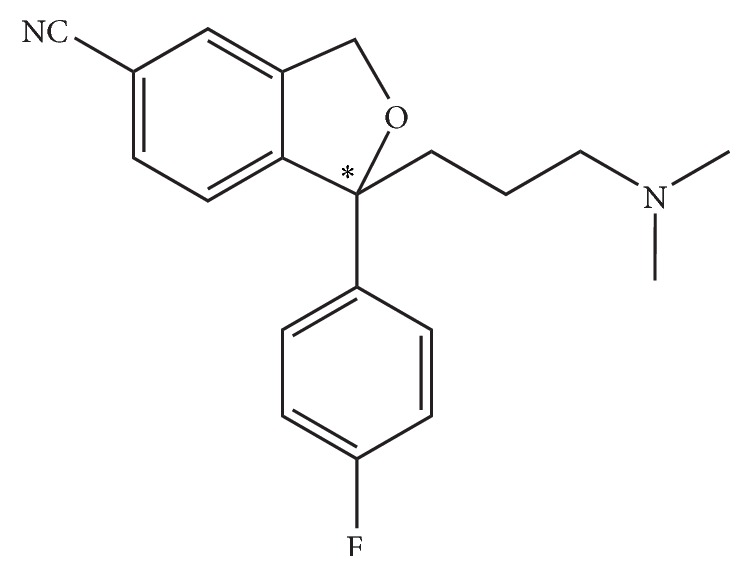
Structure of (*R, S*)-citalopram.

Citalopram (CIT), chemically 1-(3-(dimethylamino) propyl)-1-(4-fluorophenyl)-5-phthalancarbo-nitrile, is a chiral drug, widely used to treat depression and panic anxiety [1]. Its structure is shown in Figure 1. Citalopram possesses one asymmetric carbon, giving a pair of enantiomers, (*S*)-citalopram and (*R*)-citalopram. The *S* form citalopram has been proven to have much higher medicinal potency than the racemic form due to pharmacologically inactive and even counteracting nature of the *R* form citalopram [2]. The *S* form citalopram, escitalopram, is commercially available around the world under the brand name Lexapro or Cipralex [3]. According to the guidelines launched by the U.S. Food and Drug Administration (FDA) in 1992, all drugs with chiral centers are recommended to be marketed in the form of pure enantiomer, and other enantiomers without any medicinal effects are considered as impurities [4].

In order to quantitatively determine the enantiomeric composition of citalopram, various analytical methods have been researched and developed in recent years [5–21]. Most of them involve the employment of high performance liquid chromatography (HPLC) or supercritical fluid chromatography (SFC), equipped with chiral stationary phases [5–17]. As early as in 1995 [6, 7], the pioneer work on enantiomeric separation of citalopram by HPLC was conducted by Rochat et al., using a Chiralcel OD column and an acetylated *β*-cyclobond column. Since then, many efforts were made to use a number of commercially available chiral stationary phases for analyzing enantiomeric purity of citalopram, including Chiralcel OD-R column [8], Chirobiotic V column [9–11], Chiralcel OD-H column [12], Chiral-AGP column [13], and Chiralpak AD column [14]. Most recently, Li et al. [15] explored the enantioseparation of CIT using supercritical fluid chromatography equipped with a Chiralpak AD column. The effect of organic modifiers in mobile phase, column temperature, and operation pressure was studied. Additionally, some studies were focused on the development of more efficient chiral stationary phases. For example, Zhang et al. [16] developed a copolymer chiral stationary phase derived from (*1R, 2R*)-(+)-1, 2-diphenylethylenediamine. Chen et al. [17] prepared a novel stationary phase containing two structurally similar chiral selectors synthesized from D-tartrates. These newly developed chiral stationary phases have demonstrated improved enantioselectivity and have been successfully applied on chiral separation of CIT.

Contrary to using chiral stationary phases, another viable option is to add a chiral additive into the mobile phase to form a pair of transient diastereomeric complexes, followed by chromatographic separation on an achiral column. Introducing chiral additives in mobile phase has a number of advantages over using chiral stationary phases [22, 23]. First, the method is less expensive. As compared to conventional achiral columns, chiral columns, in general, are of high price, less stability, and low capacity and have a relatively short lifetime. Second, this method offers greater flexibility. Diverse achiral columns and chiral additives can be used. Moreover, the approach based on chiral mobile phases can work in both a normal phase and a reversed phase operation mode.

Cyclodextrin and its derivatives [24, 25] have been recognized as the most prevalent chiral mobile phase additives due to the nature of being nontoxic, nonflammable, nonvolatile, stable over a wide range of pH and negligible absorption in the UV ranges broadly used in chromatographic detection. They, therefore, have been successfully applied in the enantiomeric separation of a variety of racemates reported in a review article [24], and recently, of mandelic acid derivatives [26], pantoprazole [27], cathinone and amphetamine derivatives [28], phenyllactic acid [29], 2-arylpropionic acid [30], sertraline [31], beta-carboline alkaloids [32], and amlodipine [33].

**Figure 2:**
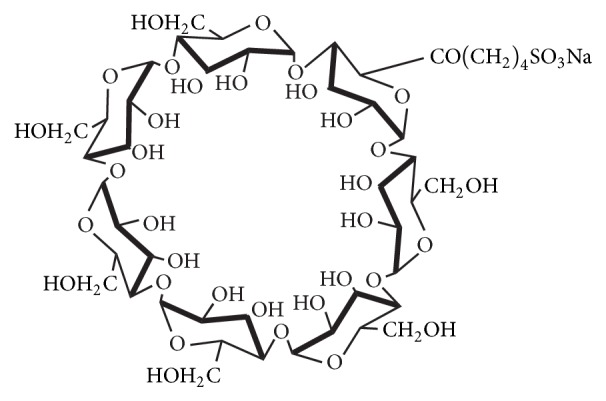
Structure of SBE-*β*-CD.

The goal of this research work is to explore the use of sulfobutylether-*β*-cyclodextrin (SBE-*β*-CD), a newly developed cyclodextrin derivative as a chiral mobile phase additive to enantioseparation citalopram on a silica-based reversed phase C18 column. SBE-*β*-CD was considered as the chiral mobile phase additive in our work because of its favorable structure and modified physicochemical properties, relative to native cyclodextrin. As illustrated in Figure 2, the presence of sulfobutyl group results in stronger acidity, leading to an increased solubility in water, while the cavity structure is well preserved. This enables SBE-*β*-CD in mobile phase to readily form complexes with the guest molecule, citalopram. From the steric structure point of view, the sulfobutyl group may partially block the mouth of the cyclodextrin and consequently alter the complexation behavior, thus resulting in variation of chiral selectivity. Our study indicated that SBE-*β*-CD was an effective chiral mobile phase additive, exhibiting high enantioselectivity ability. To the best of our knowledge, this work is the first attempt of employing sulfobutylether-*β*-cyclodextrin (SBE-*β*-CD) as a chiral mobile phase additive to separate citalopram on an achiral column instead of chiral stationary phases, offering a promising alternative for citalopram enantiomers quantitation.

## 2. Materials and Methods

### 2.1. Chemicals

SBE-*β*-CD (>98%) was purchased from Shandong Binzhou Zhiyuan Biotechnology Company Ltd. (Binzhou, China). Racemic citalopram hydrobromide and escitalopram oxalate were provided by HuaHai Pharma (Taizhou, China) with purity larger than 99.0%. Methanol and acetonitrile are of HPLC grade. Methanol was purchased from L.B. (Suzhou, China) and acetonitrile was from J&K (Beijing, China). Sodium dihydrogen phosphate (analytical grade) was purchased from L.F. Fine-Chemical (Shanghai, China) and phosphoric acid (analytical grade) from National Medicine (Shanghai, China). Standard buffers of pH was from National Medicine (Shanghai, China) and purity water from Wahaha (Hangzhou, China).

### 2.2. Instrumentation and Chromatographic Conditions

The HPLC system (Hegong Instrument, Shanghai, China) consisted of a Vertex STI P5000 pump, a STI UV detector (wavelength range of 190–700 nm), 10 *μ*L sample loop (Rheodyne), and a 7725i sampler. A Leici pHS-25 pH (Shanghai, China) meter equipped with an E-201-C combined glass electrode was used for pH measurements. It was calibrated using standard buffers of pH values of 4.01, 6.86, and 9.18. The chromatographic and the integrated data were collected using a N3000 data-acquiring software based on a window XP computer system. Before being delivered to the HPLC system, the mobile phase was filtered through 0.45 *μ*m PTFE filters and was degassed. Chromatographic separation was conducted on a Hedera ODS-2 silica gel C_18_ column (250 mm × 4.6 mm × 5.0 *μ*m, Hanbang, China) in the reversed phase operating mode, with a mobile phase consisting of aqueous buffer (sodium dihydrogen phosphate and SBE-*β*-CD), methanol, and acetonitrile with the volumetric ratio of 21 : 3 : 1. The pH value of aqueous buffer was adjusted by using dilute orthophosphoric acid. The measurements were carried out in an isocratic elution mode at ambient temperature with a flow rate of 1.0 mL/min, an injection volume of 10 *μ*L, and UV detection at 240 nm. Each experiment was run in duplicate and each solution was prepared freshly.

### 2.3. Preparation of Citalopram Free Base

The conversion of citalopram hydrobromide into racemic citalopram free base was carried out as reported in the literature [34, 35]. 10 g of citalopram hydrobromide was suspended in 20 mL water and 20 mL dichloromethane. A solution of sodium hydroxide (1.3 M) was slowly added until pH value of 10 was reached. The mixture was stirred for 30 min and settled for additional 30 min, and then the two resulting phases were separated. The organic phase was washed three times using 30 mL water each time and then concentrated on a rotary evaporator to obtain translucent oil, citalopram-free base in dichloromethane. The rest of the solvent in free base was further removed through a vacuum oven for 1 h at 80°C, giving free base citalopram in white crystalline. The crude free base crystals were further purified by recrystallization. The melting point of the final product was 90–93°C, determined by a melting point apparatus. The conversion of escitalopram oxalate into (*S*)-CIT free base was performed in the same way.

### 2.4. Samples Preparation

There was no special sample preparation required. Typically, 0.0035 g racemic citalopram was weighed and dissolved in 50 mL mobile phase solution in a volumetric flask, with the aid of ultrasonication, giving CIT concentration of 0.22 mM.

### 2.5. Determination of the Elution Order of* (R)*-CIT and* (S)*-CIT

An aliquot of racemic CIT and (*S*)-CIT was dissolved in the mobile phase, and 10 *μ*L of the mixture was submitted to chromatographic analysis under the above-mentioned conditions. Examining the two elution peak areas, the later peak had a larger area, corresponding to (*S*)-CIT.

## 3. Results and Discussion

### 3.1. Effect of the pH of Aqueous Buffer

**Figure 3:**
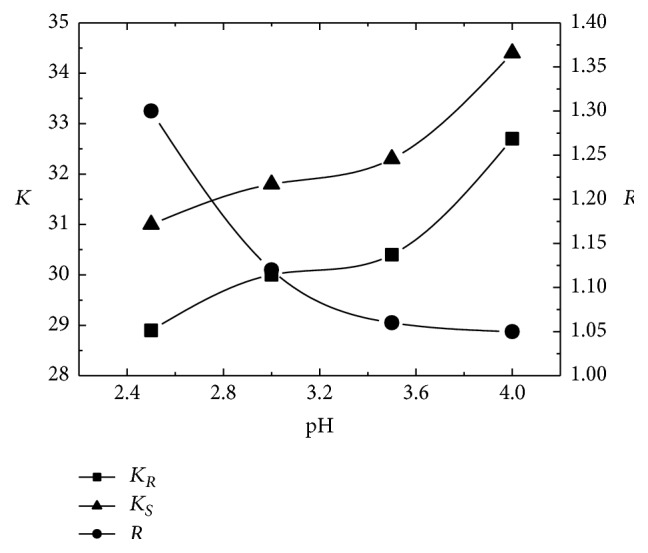
Effect of the pH of aqueous buffer. Mobile phase: aqueous buffer/methanol/acetonitrile of 21 : 3 : 1 (v/v/v); 5 mM sodium dihydrogen phosphate, 12 mM SBE-*β*-CD, flow rate of 1.0 mL/min, detection wavelength of 240 nm, and temperature of 25°C. *K*_*R*_ and *K*_*S*_, retention factors of (*R*)-CIT and (*S*)-CIT, respectively; *R*, resolution.

In chromatographic separation, the pH value of mobile phase is one of the most important factors influencing the resolution of enantiomers. In this work, the effect of the pH value on enantioseparation of CIT was first examined. The pH value was set in the range of 2.5–4.0 based on the following considerations. A very low pH value promotes the protonation of SBE-*β*-CD while it negatively impacts the packing materials in HPLC column. However, under a neutral (pH of 7) condition, dissociation of sulfobutyl group is not complete, leading to less diastereomeric complex formation. Results are illustrated in Figure 3. Here, *K*_*R*_ and *K*_*S*_ represent the retention factors of (*R*)-CIT and (*S*)-CIT, respectively, and *R* is the resolution.

As seen from Figure 3, the pH of aqueous buffer strongly affected the resolution of CIT enantiomers. The resolution of CIT enantiomers decreased from 1.30 to 1.05 when the pH was increased from 2.5 to 4.0, while the retention factors increased with the increase of pH. The pKa of CIT is about 9.5, indicating a weak alkaline nature. Under the pH range examined, CIT was almost completely protonated in the aqueous buffer; thus, CIT mainly existed as cations. Alternatively, SBE-*β*-CD possessed a sulfobutyl group, and the degree of dissociation was significantly impacted by the pH of aqueous buffer. The lower pH led to a high proportion of undissociated sulfobutyl group, which may alter the interactions between SBE-*β*-CD and CIT enantiomers. As observed, low pH values were favorable to the resolution of CIT enantiomers. However, too low pH of aqueous buffer may have adverse effects on column and chromatographic system employed. In this study, the pH value was chosen be 2.5 for the subsequent work.

### 3.2. Effect of the Concentration of SBE-*β*-CD

The effect of SBE-*β*-CD concentration on enantioseparation was investigated under the pH of 2.5, and the results are summarized in Table 1. A range of SBE-*β*-CD concentrations from 4 mM to 16 mM was tested. Obviously, the concentration of SBE-*β*-CD has a slight impact on the resolution but a significant effect on the retention time. The higher the SBE-*β*-CD concentration, the shorter the retention time. Considering that the pressure of column would increase with the increase in SBE-*β*-CD concentration, the SBE-*β*-CD concentration of 12 mM was chosen as a compromise between a good resolution and a relatively short retention time.

### 3.3. Effect of the Concentration of Sodium Dihydrogen Phosphate

**Figure 4:**
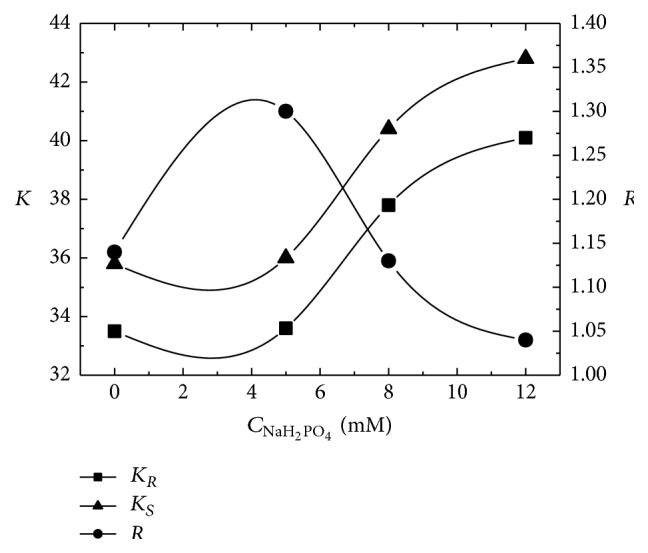
Effect of the concentration of sodium dihydrogen phosphate. Mobile phase: aqueous buffer/methanol/acetonitrile of 21 : 3 : 1 (v/v/v), C_NaH_2_PO_4__, concentration of sodium dihydrogen phosphate in aqueous buffer of 0, 5, 8, and 12 mM, 12 mM SBE-*β*-CD, pH of 2.5, flow rate of 1.0 mL/min, detection wavelength of 240 nm, temperature of 25°C, *K*_*R*_ and *K*_*S*_, and retention factors of (*R*)-CIT and (*S*)-CIT, respectively; *R*, resolution.

The sodium dihydrogen phosphate concentration affects the stability of inclusion complexes formed between the chiral additive and analyte to certain degree [25, 36] (hereby, (*R*)-CIT·SBE-*β*-CD and (*S*)-CIT·SBE-*β*-CD), further influencing the resolution and retention time. The effect of concentration of sodium dihydrogen phosphate on enantioseparation was examined by varying the concentration of sodium dihydrogen phosphate from 0 to 12 mM, and the results are shown in Figure 4. It is clearly shown that a concentration of 5 mM of sodium dihydrogen phosphate was the best, giving a high resolution and low retention factors under our experimental conditions.

### 3.4. Effect of the Composition of the Mobile Phase

The mobile phase composition was found to influence the retention time and resolution of CIT enantiomers. Further investigation was conducted by varying the volumetric ratio of aqueous buffer, methanol, and acetonitrile under the following conditions: pH of 2.5, SBE-*β*-CD concentration of 12 mM, and sodium dihydrogen phosphate concentration of 5 mM. As shown in Table 2, the variation of organic modifiers' content has substantial impact on enantioseparation of CIT. Upon increasing the proportions of methanol or acetonitrile, both retention time and resolution decreased accordingly. In order to obtain satisfactory resolution and retention time, the volume ratio of 21 : 3 : 1 was selected.

### 3.5. Effect of the Column Temperature

The effect of column temperature on the resolution of CIT enantiomers was studied in a range of 20°C to 35°C. When the column temperature was increased, the chromatographic parameters including retention factor and resolution decreased accordingly as seen in Table 3. To achieve a fair balance between higher resolution and lower retention time, 25°C was chosen as the operating temperature.

**Figure 5:**
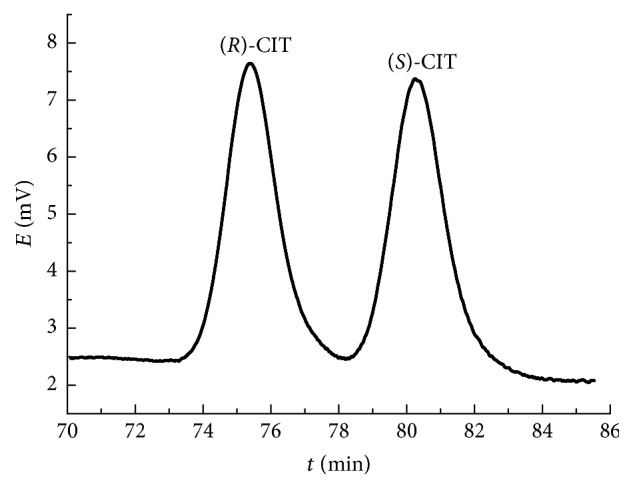
Typical chromatogram of CIT enantiomers. Mobile phase: aqueous buffer/methanol/acetonitrile of 21 : 3 : 1 (v/v/v), 5 mM sodium dihydrogen phosphate, 12 mM SBE-*β*-CD, pH of 2.5, flow rate of 1.0 mL/min, detection wavelength of 240 nm, temperature of 25°C, and injection of 10 *μ*L and 0.22 mM of racemic CIT.

In summary, under the experimental scope in this study, the optimal operating conditions were determined to be as follows: mobile phase containing a mixture of aqueous buffer, methanol, and acetonitrile with a ratio of 21 : 3 : 1 (v/v/v); the concentrations of sodium dihydrogen phosphate and SBE-*β*-CD in aqueous buffer of 5 mM and 12 mM, respectively; pH of 2.5; flow rate of 1.0 mL/min; detection wavelength of 240 nm; column temperature of 25°C; injection volume of 10 *μ*L; and racemic CIT concentration of 0.22 mM. The typical chromatogram of CIT enantiomers is shown in Figure 5.

### 3.6. Method Validation

The proposed method was validated through the examination of precision, linearity, limit of detection (LOD), and limit of quantitation (LOQ).

#### 3.6.1. Precision

Precision was evaluated by determining the relative standard deviations (RSDs). The sample solution with a known concentration of racemic CIT (0.03 g/L) was prepared. Sequentially, six sample solutions were injected to the column under the same chromatographic conditions. The RSDs of retention time were 1.9% for (*R*)-CIT and 2.3% for (*S*)-CIT, respectively; the RSDs of peak areas were 1.5% for (*R*)-CIT and 2.8% for (*S*)-CIT, respectively, and the RSD of resolution was 2.5%.

#### 3.6.2. Accuracy

The accuracy was evaluated by determining the percent recovery through standard additions. As shown in Table 4, the recovery of (*R*)-CIT and (*S*)-CIT is 93.8% and 93.7%, respectively, demonstrating that the reported method is accurate for analyzing (*R*)-CIT and (*S*)-CIT enantiomers.

#### 3.6.3. Linearity

Linearity was examined by conducing triplicate injections at six concentration levels of racemic CIT (ranging from 0.01 to 0.06 g/L). Calibration curves of each enantiomer were linear over the tested concentration range. Regression equations (the peak area versus the concentration of each enantiomer in g/L) showed fairly good linearity with regression coefficients (*r* ^2^) of 0.9988 for (*R*)-CIT and 0.9995 for (*S*)-CIT, being presented as follows:

(1) y=1.85760×107CR+3774r2=0.9988,y=1.93443×107CS−1755r2=0.9995.

#### 3.6.4. Limit of Detection (LOD)

Limit of detection (LOD) is defined as the lowest analyte concentration that can be detected, giving accuracy within ±15% (bias) of the nominal value, and 15% of interassay precision. Limit of quantitation (LOD) is defined as the lowest analyte concentration that can be detected to produce a significant response. Both of them are important parameters to assess an analytical method. In this work, LOD and LOQ were calculated using signal/noise (*S*/*N*) ratio method. LOD was taken as the concentration of analyte in which the *S*/*N* was 3 while LOQ was taken as the concentration of analyte where *S*/*N* was 10. The LODs of (*R*)-CIT and (*S*)-CIT were found to be 0.070 *μ*g/mL and 0.076 *μ*g/mL, respectively. LOQs were 0.235 *μ*g/mL for (*R*)-CIT and 0.254 *μ*g/mL for (*S*)-CIT, respectively.

## 4. Conclusions

This paper describes a new and reliable method for CIT enantioseparation by HPLC, using SBE-*β*-CD as a chiral mobile phase additive. Sulfobutylether-*β*-cyclodextrin was used for the first time as a chiral mobile phase additive for determining the enantiomeric composition of racemic citalopram, rather than involving costly chiral stationary phases.

The chromatographic conditions were optimized, and a fairly good enantioseparation was obtained under the following conditions: aqueous buffer consisting of 5 mM sodium dihydrogen phosphate and 12 mM SBE-*β*-CD with the pH of 2.5, the ratio of aqueous buffer, methanol, and acetonitrile of 21 : 3 : 1 (v/v/v), and the column temperature of 25°C. However, it is noteworthy that the retention time was 75 minutes, relatively long, which might undermine the efficiency and robustness of this newly developed method. More research is needed to improve retention time, possibly through changing organic modifiers and/or adding a second chiral selector.

## Figures and Tables

**Table 1:** Effect of the concentration of SBE-*β*-CD in aqueous phase.

*C*_SBE-β-CD_ (mM)	*K*_*R*_	*K*_*S*_	*R*	*α*
4	52.5	55.7	1.27	1.06
8	42.0	44.7	1.30	1.06
12	33.6	36.0	1.30	1.07
16	32.0	34.0	1.31	1.06

Mobile phase: aqueous buffer/methanol/acetonitrile of 21 : 3 : 1 (v/v/v), concentration of SBE-*β*-CD in aqueous buffer of 4, 8, 12, and 16 mM, 5 mM sodium dihydrogen phosphate, pH of 2.5, flow rate of 1.0 mL/min, detection wavelength of 240 nm, temperature of 25°C, *K*_*R*_ and *K*_*S*_, and retention factors of (*R*)-CIT and (*S*)-CIT, respectively; *R*, resolution, and *α*, separation factor.

**Table 2:** Effect of the composition of mobile phase.

*V*_water_ : *V*_methanol_ : *V*_acetonitrile_	*K*_*R*_	*K*_*S*_	*R*	*α*
22 : 3 : 1	39.9	42.7	1.32	1.07
21 : 3 : 1	28.3	30.3	1.30	1.07
20 : 3 : 1	27.6	29.4	1.24	1.06
21 : 3 : 2	15.3	16.2	1.07	1.06
21 : 3 : 0	72.1	73.4	1.40	1.02

Mobile phase: aqueous buffer/methanol/acetonitrile with various ratios, 12 mM SBE-*β*-CD, 5 mM sodium dihydrogen phosphate, pH of 2.5, flow rate of 1.0 mL/min, detection wavelength of 240 nm, temperature of 25°C, *K*_*R*_ and *K*_*S*_, and retention factors of (*R*)-CIT and (*S*)-CIT, respectively; *R*, resolution, and *α*, separation factor.

**Table 3:** Effect of the column temperature.

*T* (°C)	*K*_*R*_	*K*_*S*_	*R*	*α*
20	34.4	37.2	1.33	1.08
25	31.5	33.8	1.30	1.07
30	28.1	30.0	1.20	1.07
35	24.1	25.6	1.12	1.06

Mobile phase: aqueous buffer/methanol/acetonitrile of 21 : 3 : 1 (v/v/v), concentration of SBE-*β*-CD in aqueous buffer of 4, 8, 12, and 16 mM, 5 mM sodium dihydrogen phosphate, pH of 2.5, flow rate of 1.0 mL/min, detection wavelength of 240 nm, temperature of 25°C, *K*_*R*_ and *K*_*S*_, and retention factors of (*R*)-CIT and (*S*)-CIT, respectively; *R*, resolution, and *α*, separation factor.

**Table 4:** Test of the accuracy of (*R*)-CIT and (*S*)-CIT by standard additions.

Concentration (*μ*g/mL)				Recovery (%)
Sample	Standard	Nominal	Calculated	
(*R*)-CIT	0, 3.95, 7.91, 11.9, 15.8	9.88	9.27	93.8
(*S*)-CIT	0, 3.95, 7.91, 11.9, 15.8	9.88	9.26	93.7

Mobile phase: aqueous buffer/methanol/acetonitrile of 21 : 3 : 1 (v/v/v), 5 mM sodium dihydrogen phosphate, 12 mM SBE-*β*-CD, pH of 2.5, flow rate of 1.0 mL/min, detection wavelength of 240 nm, temperature of 25°C, and injection of 10 *μ*L and 0.22 mM of racemic CIT.
